# Highly Sensitive and Selective Sol-Gel Spin-Coated Composite TiO_2_–PANI Thin Films for EGFET-pH Sensor

**DOI:** 10.3390/gels8110690

**Published:** 2022-10-26

**Authors:** Nur Syahirah Kamarozaman, Nurbaya Zainal, Aimi Bazilah Rosli, Muhammad Alhadi Zulkefle, Nik Raikhan Nik Him, Wan Fazlida Hanim Abdullah, Sukreen Hana Herman, Zurita Zulkifli

**Affiliations:** 1 Integrated Sensors Research Group, School of Electrical Engineering, College of Engineering, Universiti Teknologi MARA (UiTM), Shah Alam 40450, Selangor, Malaysia; 2 NANO-ElecTronic Centre, School of Electrical Engineering, College of Engineering, Universiti Teknologi MARA (UiTM), Shah Alam 40450, Selangor, Malaysia; 3 Microwave Research Institute, Universiti Teknologi MARA (UiTM), Shah Alam 40450, Selangor, Malaysia

**Keywords:** EGFET, sol-gel spin-coating method, TiO_2_ thin film, composite TiO_2_–PANI thin film, pH sensor

## Abstract

A highly selective and sensitive EGFET-pH sensor based on composite TiO_2_–PANI had been developed in this work. A sol-gel titanium dioxide (TiO_2_) and the composite of TiO_2_ with semiconducting polyaniline (PANI) were deposited using a simple spin-coating method on an indium tin oxide (ITO) substrate. The films have been explored as a sensing electrode (SE) of extended gate field-effect transistor (EGFET) for pH applications in the range of pH 2 to 12. The pH sensitivities between TiO_2_, TiO_2_–PANI bilayer composite, and TiO_2_–PANI composite thin films were discussed. Among these, the TiO_2_–PANI composite thin film showed a super-Nernstian behavior with high sensitivity of 66.1 mV/pH and linearity of 0.9931; good repeatability with a standard deviation of 0.49%; a low hysteresis value of 3 mV; and drift rates of 4.96, 5.54, and 3.32 mV/h in pH 4, 7, and 10, respectively, for 6 h. Upon applying the TiO_2_–PANI composite as the SE for nitrate measurement, low sensitivity of 12.9 mV/dec was obtained, indicating that this film is a highly selective sensing electrode as a pH sensor. The surface morphology and crystallinity of the thin films were also discussed.

## 1. Introduction

An ion-sensitive field-effect transistor (ISFET) technology has been reported as an alternative to the glass electrode due to its advantages such as small size and solid-state nature, mass fabrication, short response time, and low output impedance [1]. However, the ISFET fabrication process is very complicated and requires high manufacturing technology [2]. Thus, the extended gate field-effect transistor (EGFET) was introduced by Spiegel in 1983 as an improved version of the ISFET. The EGFET consists of a FET and sensing electrode that is separated into two parts, in which only the sensing electrode is in contact directly with the electrolyte while the FET is being isolated. Due to its low cost, ease of fabrication, excellent responsiveness, ease of operation, lightweight, and external temperature stability, the EGFET was chosen for detection applicable for disposable devices.

Sensing features such as sensitivity, selectivity, response time, reusability, long-term stability, and cost are important in developing a sensor. Thus, the selection and design of the sensing material used as a sensing electrode are significant with regard to sensor performance. Numerous studies have been carried out to develop EGFET-pH sensors based on different sensing electrodes. pH sensors based on metal oxides such as titanium dioxide (TiO_2_) [3,4], tin oxide (SnO_2_) [5], zinc oxide (ZnO) [6,7], ruthenium oxide (RuO_2_) [8], nickel oxide (NiO) [9], copper oxide (CuO) [10], tantalum oxide (Ta_2_O_5_) [11], and others have been studied owing to their high sensitivity to hydrogen ions and a high degree of accuracy. Among these, TiO_2_ is a promising material owing to its superior chemical stability, nontoxicity, relatively inexpensive costs, and easy fabrication [3,4]. Cheng Chen et al. [3] developed a porous TiO_2_ thin film using a chemical etching method with a reaction time ranging from 15 to 240 min. They found that a solution concentration of 1 M and reaction time of 120 min gave high sensitivity of 54.13 mV/pH, a response time of 18.1 s in pH 4 to 12, an acceptable drift of 9.29 mV/h, and a hysteresis width of 11.4 mV. H. Khizir et al. [12] used the hydrothermal method to fabricate TiO_2_ nanorods at a hydrothermal reaction temperature of 170 °C for 6 h. The sensor exhibited high sensitivity of 78.25 mV/pH and a large linearity of 99.27% with good repeatability of 0.23% in a sensing range of pH 2 to 12. The device also showed good stability, reliability, and a lower hysteresis value of 9.1 mV. Yao et al. [13] reported a sol-gel TiO_2_ thin film annealed at 200 °C for 30 min showed the highest sensitivity of 79.9 µA/pH. Based on these literatures, TiO_2_-based films are considered good for pH sensing layers in strong acid and alkali solutions. However, as reported by Manjakkal et al. [14], TiO_2_ possesses high sheet resistance because of its intrinsic property. Thus, modification of TiO_2_ surface or doping with metallic ions is suggested to increase the conductivity of the films and improve the sensing performance of the device.

Conducting polymers can be considered to improve the sensing performance of the devices. Some of these MOx-based sensors have a slow response to neutral and basic solutions [14], and one of the alternative methods is to mix metal oxide with conductive material, which acts as a path of ion transfer [14]. Polymers such as polyaniline (PANI) have been studied as part of sensing electrodes due to their relatively air-stable, high electrical conductivity; good environmental, chemical, and electrical stability; and easy synthesis. Composite of metal oxide–PANI shows potential in pH application. For example, Mazzara et al. [15] fabricated PANI thin films with reduced graphene oxide using the potentiostat deposition technique. The device showed good sensitivity of 62.3 mV/pH and reproducibility of 3.8%. Wang et al. [16] developed a pH sensor with temperature compensation based on copper oxide–polyaniline modification. The device exhibited high sensitivity and linearity (0.288 mA/pH, R^2^ = 0.9998), high accuracy, and response time of less than 4 s. Moreover, Su et al. [17] reported a graphene and polyaniline composite film using electrochemical polymerization on ITO glass. The device showed high sensitivity of 51.1 mV/pH in a wide detection range from pH 1 to 11 and good reproducibility of five fabricated sensors. However, they found that the sensitivity of the same devices decreased after two weeks, which was caused by the conductivity decrease in the composite film. As suggested by these authors, the dissolved oxygen and other oxidants could destroy the polymer backbone or weaken the cooperation of polymer and amino-functionalized graphene, leading to the reduction of the effective area on the electrode surface.

Several methods have been reported to deposit these thin films to be applied as pH sensors, such as sputtering [9,18], screen-printing [19,20], hydrothermal [12], spray pyrolysis [21], sol-gel [13,22], and electron beam evaporation [23]. Among these, the sol-gel method is cost-effective, is easy to prepare, has low reaction temperature, can adapt to various substrates, and makes it possible to produce a homogenous composite with high purity [22,24]. However, the sol-gel method has its drawback such as difficulty in controlling the precursor concentration and post-deposition annealing process that will lead to significant impact on the characteristics and performance of the films [22]. For these reasons, many researchers focused on better solution by optimizing the sol-gel spin-coating method in order to produce good quality films and able to create thinner films [24].

Our study aimed to develop a stable and reliable soil sensor. To achieve that, we explored the application of sol-gel spin-coated TiO_2_-based thin films for soil pH sensors while considering the varieties of ions existing in the soil environment. This paper discussed the sensing performance of the EGFET-pH sensor based on TiO_2_, TiO_2_–PANI bilayer composite, and TiO_2_–PANI composite thin films as the sensing electrodes (SEs). The indium tin oxide (ITO) coated glass was used as the substrate since the sensing electrode of an EGFET must be made of conductive material to allow the transmission of sensing signals [25]. All samples were measured for pH sensing performance and tested for sensor selectivity by applying the same SE for nitrate measurement.

## 2. Result and Discussion

### 2.1. Physical Properties

#### 2.1.1. Surface Morphology

**Figure 1:**
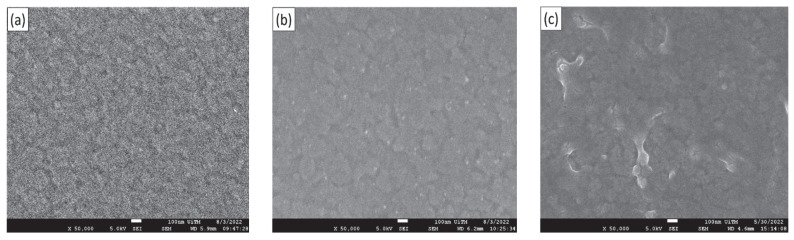

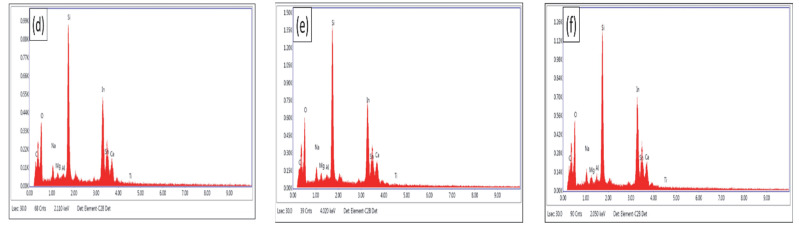
FESEM images and EDX spectra of (**a**,**d**) TiO_2_ thin film; (**b**,**e**) TiO_2_–PANI bilayer composite; and (**c**,**f**) TiO_2_–PANI composite thin films.

The sensing electrode surface morphology significantly influences its sensing performance [19]. Thus, the surface morphology of the prepared SE was characterized using field-emission scanning electron microscopy (FESEM) (JEOL JSM-7600F) with an operating voltage of 5 keV and a magnification of 50 k. Figure 1 shows the FESEM images of the TiO_2_, TiO_2_–PANI bilayer composite, and TiO_2_–PANI composite thin films. Both the TiO_2_ and TiO_2_–PANI bilayer composite thin films (Figure 1a,b) relatively showed a smooth surface morphology. Meanwhile, a rough surface with undistributed agglomerate particle size was observed for the TiO_2_–PANI composite thin film (Figure 1c). The results showed that the prepared TiO_2_–PANI composite is not well-dispersed, which then might contribute to the non-uniform surface structure of the films. This is also in agreement with a previous study in which the agglomerated particles on the surface of TiO_2_–PANI composite thin films were due to the increase in the concentration of TiO_2_ [26,27]. Additionally, the study revealed that the non-uniform surface of the composite thin films induced a high surface area and hence fitted well for sensor application. The elemental analysis of these thin films was carried out by energy-dispersive X-ray spectroscopy (EDX) measurement using an acceleration voltage of 15 kV. The values of atomic (at%) for each element are summarized in Table 1. The major peaks observed in EDX results show the major elements from the ITO substrate: indium (In), tin (Sn), silicon (Si), and oxygen (O). Based on available literature [28], the peak elements of carbon (C), nitrogen (N), and oxygen (O) correspond to PANI materials. However, in this study, the N peak was not detected by the EDX analysis for both composite thin films. The same behavior was reported by Libuse et al. [29], where they suggested that the light elements such as C and N are not suitable for EDX analysis because these elements do not produce continuum intensity. Furthermore, the lower at% for titanium (Ti) and higher content of ITO substrate (In, Sn, Si, and O) might be due to the penetration depth through the thinner film resulting in more signals being collected from the substrate rather than the thin films. The other elements of sodium (Na), magnesium (Mg), aluminum (Al), and calcium (Ca) were not significant and can be ignored.

#### 2.1.2. XRD Analysis

**Figure 2:**
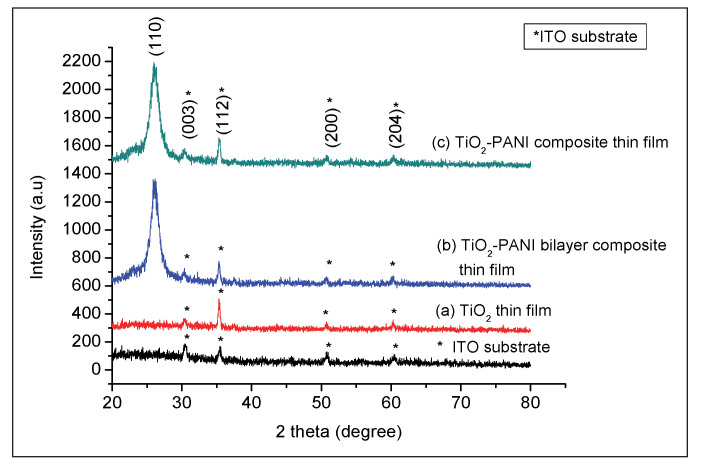
XRD pattern of (**a**) TiO_2_ thin film; (**b**) TiO_2_–PANI bilayer composite; and (**c**) TiO_2_–PANI composite thin films. The peaks of ITO substrate are indicated by solid star (*).

Figure 2a–c show the crystal structure of TiO_2_, TiO_2_–PANI bilayer composite, and TiO_2_–PANI composite thin films using an X-ray diffractometer (Rigaku Ultima IV), respectively. The XRD patterns were recorded in the range of 2θ from 20° to 80° with a step width of 0.02° and CuKα emission line (λ = 1.54060 nm, 40 kV and 40 mA). A significant peak was observed for both composite thin films (Figure 2b,c) at 2-theta (2θ) = 26.08°, which corresponds to (110) orientation. Based on the available literature [30], this orientation belongs to the PANI material. Several minor peaks were observed at 37.81°, 53.92°, and 62.75°, which corresponded to Miller indices of (112), (200), and (204), respectively, for all samples that include TiO_2_ thin films and ITO substrates. The peaks corresponded to the anatase phase, referring to the International Centre for Diffraction Data (ICDD card no. 01-086-1156). Additionally, another peak was observed at 30.55° for all samples corresponding to the (003) orientation, indicating ITO material. In this study, (101) orientation was not observed in the TiO_2_ thin films. Similar observation was also found in the previous study and claimed that higher annealing temperature is compulsory to attain such orientation [31]. Moreover, the difference in the crystalline structure of these films is also attributed to the nature of the substrate.

### 2.2. pH Sensor Performances

#### 2.2.1. pH Sensitivity

**Figure 3:**
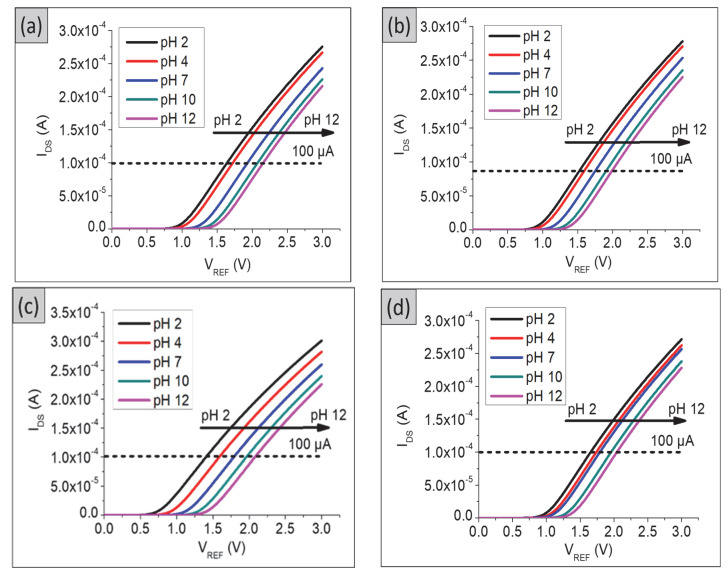
Transfer characteristics (I_DS_–V_REF)_ in linear region for (**a**) TiO_2_ thin film, (**b**) TiO_2_–PANI bilayer composite thin film, (**c**) TiO_2_–PANI composite thin film, and (**d**) ITO substrate.

The transfer characteristics (I_DS_–V_REF_) in the linear region for the TiO_2_ and composite TiO_2_–PANI thin films are shown in Figure 2. The reference electrode voltage ranged from 0 V to 3 V, and the sensing electrode was measured in a pH buffer solution with a pH range of 2 to 12. The drain voltage of the sensing electrode was kept constant at 0.1 V. Figure 3 shows that as the pH value increased, the threshold voltages moved from the left to the right (hydrogen ions concentration decreased).

The site binding models, in which the surface voltage of an oxide layer fluctuates with the pH of an electrolyte solution, can be used to explain the pH process [12,32]. The threshold voltage shift is caused by a change in the surface potential at the interface between the sensing electrode and the electrolyte caused by the sensing electrode being exposed to an electrolyte solution. The following is an expression for the surface potential voltage (*Ψ_o_*) between the oxide film and electrolyte:

(1) $$2.303\;\left(pH_{pzc}-pH\right)=\frac{q\Psi_{o}}{kT}+sinh^{-1}\;(\frac{q\Psi_{o}}{kT}.\;\frac{1}{\beta })$$

where $pH_{pzc}$ is the pH value at the point of zero charges, *q* is the electron charge, *k* is the Boltzmann constant, *T* is the absolute temperature, and *β* is the sensitivity parameter.

In general, the surface of the oxides is composed of hydroxyl groups, ${\mathrm{OH}}^{-}$. Thus, when the metal oxide surface comes into contact with a solution, the surface reaction could be protonated in acidic or deprotonated ions in an alkaline solution, thus leaving positively or negatively charged on the surface of the sensing electrodes.

**Figure 4:**
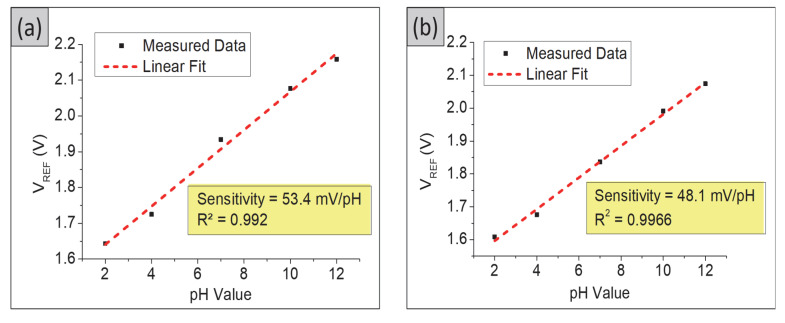

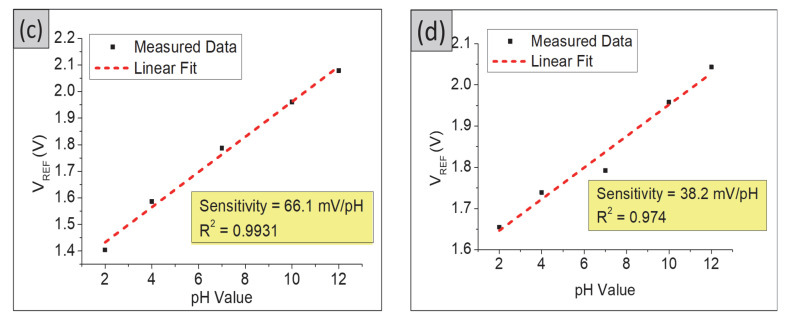
pH sensitivity in linear region for (**a**) TiO_2_ thin film, (**b**) TiO_2_–PANI bilayer composite thin film, (**c**) TiO_2_–PANI composite thin film, and (**d**) ITO substrate.

The pH sensitivity of the sensing electrodes was calculated by taking the readings at the slope of V_REF_ as a function of different pH values, at fixed I_DS_ = 100 µA, as shown in Figure 4. The sensitivity of TiO_2_ films was 53.4 mV/pH with a linearity of 0.992, whereas the TiO_2_–PANI bilayer composite showed sensitivity and linearity of 48.1 mV/pH and 0.9966, respectively. Among these SEs, the TiO_2_–PANI composite thin films showed a super-Nernstian pH response with sensitivity and linearity values of 66.1 mV/pH and 0.9931, respectively. The super-Nernstian response is considered as the pH sensitivity value over the Nernstian limit of 59 mV/pH, which could promise more precise detection in monitoring the pH levels in the blood and extracellular fluids. This super-Nernstian could be related to the enhanced proton-exchange process [23] due to the difference in fabrication conditions leading to different oxidation states of the sensing electrodes resulting in higher sensitivity. Furthermore, the use of a conducting polymer may enhance the conductivity of the composite thin film.

According to the Nernst theoretical equation (Equation (2)), the sensitivity should be approaching the theoretical value of 59 mV/pH, and the linearity should be approaching 1. The potentiometric pH sensors can be obtained by the slope of the linear regression.

(2) $$E=E^{0}=0.0592\;V\;log\frac{Q}{n}$$

where *E*, *E°*, *n*, and *Q* are the cell potential, standard cell potential, number of electrons, and reaction quotient, respectively. Thus, these fabricated samples are said to be reliable to be used in pH sensing applications since both values (sensitivity and linearity) are comparable with this equation.

**Figure 5:**
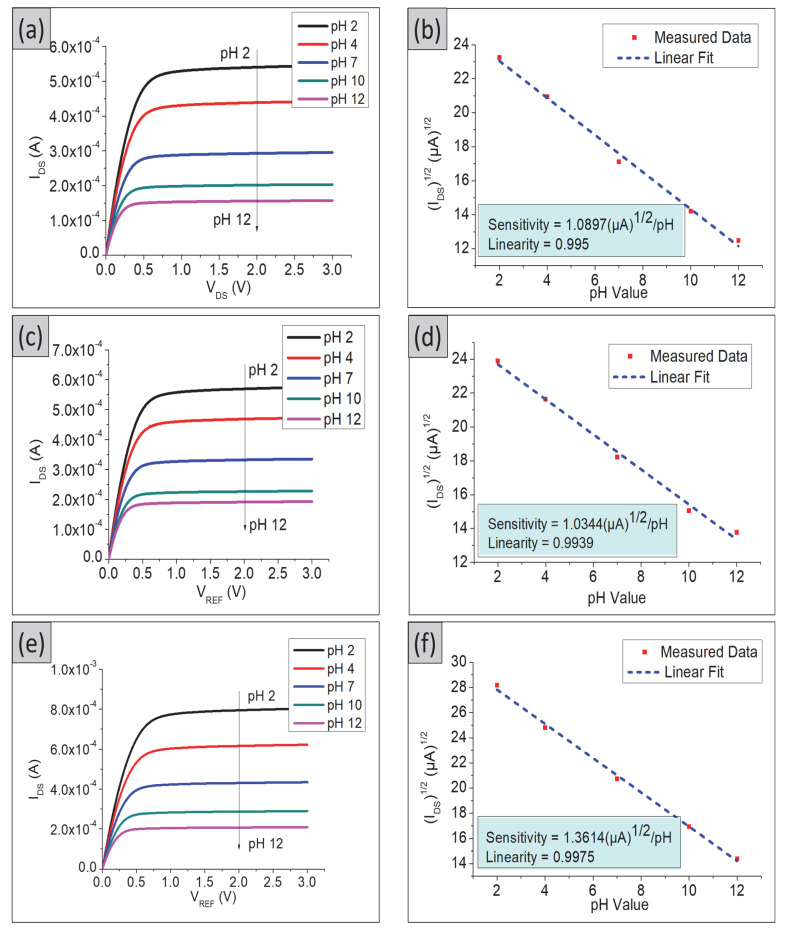

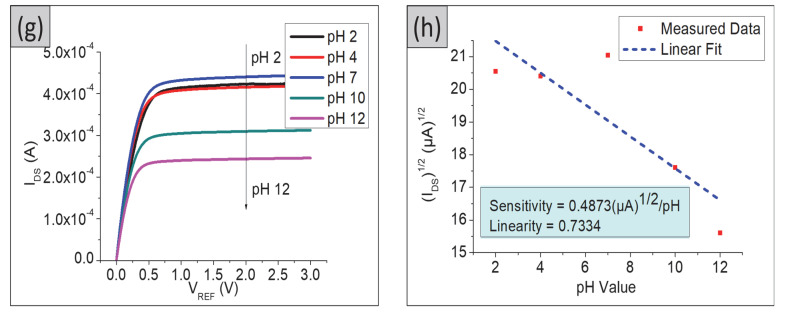
Output characteristics (I_DS_–V_DS_) and sensitivity in saturation region for (**a**,**b**) TiO_2_ thin film, (**c**,**d**) TiO_2_–PANI bilayer composite, (**e**,**f**) TiO_2_–PANI composite thin films, and (**g**,**h**) ITO substrate.

Figure 5 shows the output characteristics (I_DS_–V_DS_) for all fabricated samples in the saturation region. A channel that allows current to flow between the drain and source has been formed in this region. As can be seen in Figure 5a,c,e,g, the drain current becomes almost constant for all drain voltage for a fixed gate voltage. The saturation sensitivity was calculated by taking the readings of V_DS_ = 2 V. The pH current sensitivity for TiO_2_–PANI composite thin films was 1.3614 (µA)^1/2^/pH with a linearity of 0.9975, which is higher compared with those of TiO_2_ and TiO_2_–PANI bilayer composite thin films. The bare ITO substrate was also tested for its sensitivity and linearity in both linear and saturation regions. The pH sensitivity and linearity of the bare ITO substrate in the linear region was 38.2 mV/pH, 0.974 (Figure 3d and Figure 4d). In the saturation region, the pH sensitivity and linearity of the ITO substrate were 0.4873 (µA)^1/2^/pH and 0.7334, respectively, as shown in Figure 5g,h. A low value of pH sensitivity obtained for the ITO substrate confirmed that the higher sensitivity observed in Figure 4 and Figure 5 was from the prepared SE in this experiment.

#### 2.2.2. Selectivity Test in pH Measurement

**Figure 6:**
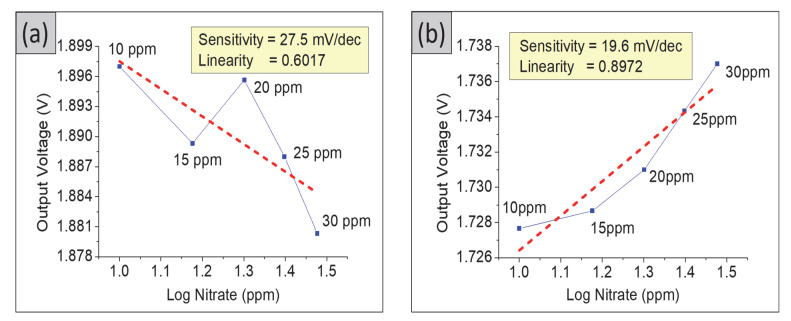

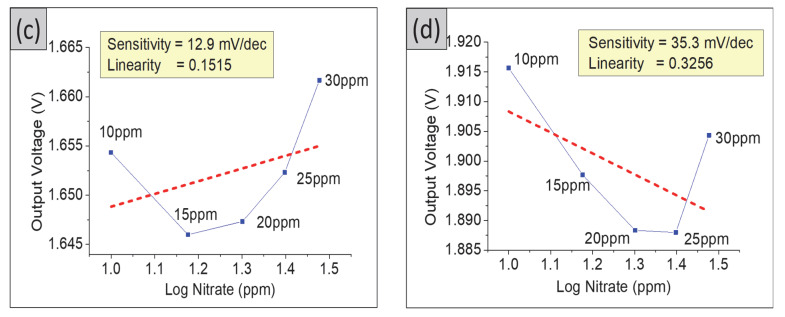
Output voltage of nitrate sensor and logarithmic nitrate concentration in the range of 10 to 30 ppm for (**a**) TiO_2_ thin film, (**b**) TiO_2_–PANI bilayer composite thin film, (**c**) TiO_2_–PANI composite thin film, and (**d**) ITO substrate.

To examine the selectivity of the SE in pH application, a solution of nitrate buffer ranging from 10 to 30 ppm was tested with the same measurement setup. The nitrate sensitivity for TiO_2_, TiO_2_–PANI bilayer composite, and TiO_2_–PANI composite thin films were 27.5, 19.6, and 12.9 mV/dec, respectively, as shown in Figure 6. Based on these findings, the TiO_2_–PANI composite thin film produced the lowest nitrate sensitivity and linearity of 0.1515. The measurement was repeated two times for the same TiO_2_–PANI composite thin film, and it was observed that the sensitivity decreased. This sample gave sensitivity and linearity values of 9.2 mV/dec, 0.1842 and 3.4 mV/dec, 0.0779, respectively. The bare ITO substrate showed a nitrate sensitivity of 35.3 mV/dec and a linearity of 0.3256. This shows that the materials of SE prepared in this experiment are unfit for nitrate-sensing measurement.

Several studies showed high sensitivity performance of EGFET-based sensors; for instance, Baumbauer et al. [33] developed a fully printed potentiometric nitrate sensor using a carbon nanotube and PVB as the sensing electrode. The device showed a sensitivity for all seven sensors of −54.1 ± 2.1 mV/dec in a linear region between 0.5 mM and 100 mM. Chaisriratanakul et al. [34] reported a polyvinyl chloride (PVC) as an ion-selective electrode for the ISFET-nitrate sensor and exhibited a high sensitivity of 56 ± 2 mV/dec in a linear range from 3 to 20 ppm. Interestingly, the TiO_2_–PANI composite SE prepared in this study showed very low nitrate sensitivity and linearity. These findings suggested the feasibility of this device for pH application only.

Most of the literatures reported on the use of sensing electrodes to be applied either in pH or nitrate application only [3,34,35,36]. However, in this work, the optimized SE was tested in both pH and nitrate applications. The high sensitivity and selectivity of the prepared TiO_2_–PANI composite thin films show a possibility to distinguish between the $H^{+}$ and ${\mathrm{NO}}_{3}^{-}$ ions, thus suggesting that this sample can be applied in the agriculture sector since both ions are present in soil.

Based on these studies, the TiO_2_–PANI composite thin film fitted well for EGFET-pH sensors due to the larger distinction values between pH and nitrate sensitivity. The device showed the highest pH sensitivity and linearity of 66.1 mV/pH and 0.9931, respectively, in contrast to the nitrate sensitivity of only 12.9 mV/dec. These findings suggested that the device was highly sensitive and selective in pH applications.

### 2.3. Stability and Reliability

#### 2.3.1. Hysteresis Measurement

**Figure 7:**
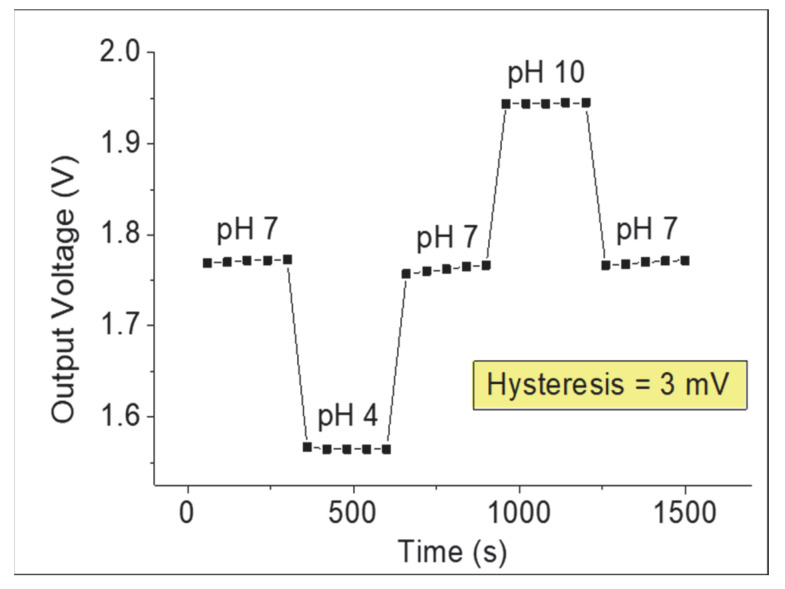
Hysteresis characteristic of composite TiO_2_–PANI thin film in pH loops of 7→4→7→10→7.

The short-term stability or hysteresis characteristics of the composite TiO_2_–PANI EGFET-pH sensor were measured by immersing the SE in alternating cycles of different pH buffer solutions with measurement loops of pH 7→4→7→10→7 for 25 min. Figure 7 shows the hysteresis characteristics for both samples. The curve was plotted by taking the slope of the reference voltage for a fixed drain current of 100 µA with different pH values. The hysteresis voltage was defined by the difference between the initial and final reference voltage at pH 7. The device showed a low hysteresis voltage of 3 mV, which indicates a good delay pH response.

#### 2.3.2. Drift Measurement

**Figure 8:**
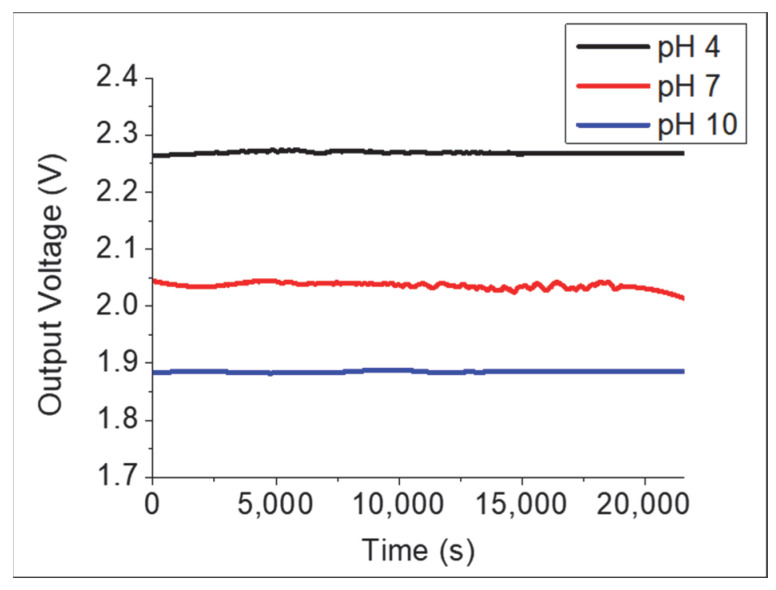
Drift characteristic of TiO_2_–PANI composite thin film.

The drift effect represents the long-term stability of electrochemical sensors. Figure 8 shows the drift curves of the TiO_2_–PANI composite thin film in pH 4, 7, and 10 buffer solutions for 6 h. The change in the reference voltage (ΔV_REF_) is defined as ΔV_REF_ = V_REF_ (t) − V_REF_ (0). The drift property is compared by calculating the drift rate (ΔV_REF_/h) [23]. The drift rate of the device was approximately 4.96, 5.54, and 3.32 mV/h for pH 4, 7, and 10, respectively.

#### 2.3.3. Repeatability

**Figure 9:**
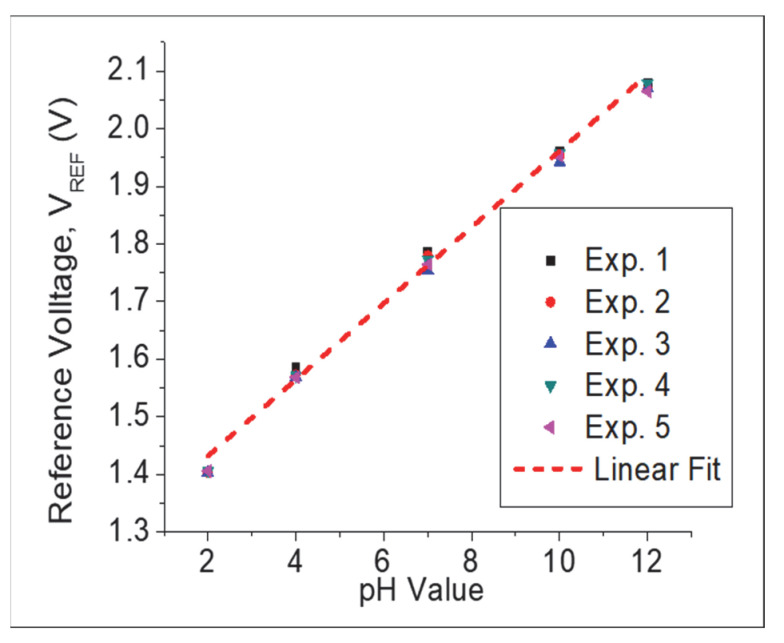
Repeatability curve of TiO_2_–PANI composite thin film.

Repeatability is defined as the usability of the same fabricated device more than once. Figure 9 shows the repeatability measurement of the TiO_2_–PANI composite thin film five times. The sensitivity values were 66.1, 65.6, 65.5, 66.5, and 65.3 mV/pH with a calculated standard deviation of 0.49%. These findings suggested that the fabricated pH sensor maintained its sensitivity, indicating good repeatability of the developed pH sensor.

## 3. Conclusions

This study described a highly selective and sensitive sol-gel spin-coated TiO_2_–PANI composite thin film as an EGFET-pH sensor. The pH sensing performance of TiO_2_, TiO_2_–PANI bilayer composite, and TiO_2_–PANI composite thin films as the SE was studied. The samples were tested in pH ranging from 2 to 12. The pH sensitivity in both linear and saturation regions was observed. All samples were tested for sensor selectivity by applying the same SE for nitrate measurement. Based on these findings, the TiO_2_–PANI composite thin film showed a high sensitivity and linearity of 66.1 mV/pH and 0.9931, respectively, for pH application, in comparison with the nitrate measurement with the lowest sensitivity of 12.9 mV/dec and linearity of 0.1515 compared with TiO_2_ and TiO_2_–PANI bilayer composite thin films. The nitrate sensitivity of the TiO_2_–PANI composite thin film decreased with sensitivity and linearity of 9.2 mV/dec and 0.1842 (second measurement), and 3.4 mV/dec and 0.0779 (third measurement), respectively. These experimental findings suggested that the prepared TiO_2_–PANI composite thin film in this study is highly sensitive and selective for pH application. To examine the stability of the TiO_2_–PANI composite as SE, the hysteresis and drift measurement were tested. For hysteresis measurement, the SE was immersed alternately in pH loops of pH 7→10→7→4→7 for 5 min each. The device showed a low hysteresis value of 3 mV, indicating that this SE is reliable to be used in pH testing. The drift test of the same SE was performed in pH 4, 7, and 10 for 6 h. The sample showed low drift rates of 4.96, 5.54, and 3.32 mV/h in pH 4, 7, and 10, respectively. In addition, the repeatability test of the same SE for five measurements was approximately the same with a calculated standard deviation of 0.49%, indicating good repeatability of the device. The prepared TiO_2_–PANI composite-based EGFET-pH sensor shows a promising candidate to be used in various chemical and biological applications, especially in the agriculture sector.

## 4. Materials and Methods

### 4.1. Materials

A commercialized indium tin oxide (ITO) substrate (size: 20 × 20 × 1.1 mm (length × width × height) and resistance: 7–10 Ω/sq) was used as a substrate. The titanium isopropoxide Ti[OCH(CH_3_)_2_]_4_ (97%) was purchased from Sigma Aldrich, Merck (Darmstadt, Germany). Glacial acetic acid (GAA) (98%) was purchased from Friendemann Schmidt. Triton X-100 (98%) was purchased from R&M Chemicals. Ethanol absolute (99.8%) was purchased from SYSTERM. The polyaniline (emeraldine base) (PANI) was purchased from Sigma-Aldrich (St. Louis, MO, USA). The pH buffer solutions (pH 2, 4, 7, 10, and 12) were purchased from Sigma Aldrich (Darmstadt, Germany). Nitrate standard solutions and a nitrate ionic strength adjuster (Nitrate ISA, Orion ionplus Application solution) were purchased from Thermo Fisher Scientific (Chelmsford, MA, USA).

### 4.2. Sensing Electrodes Preparation

**Figure 10:**
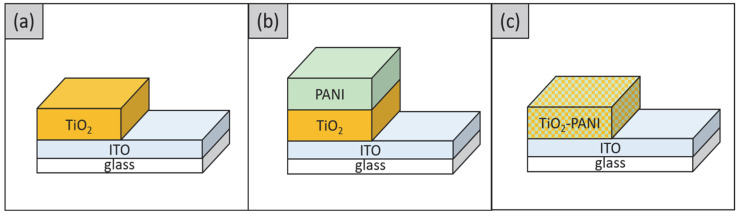
Cross-section of (**a**) TiO_2_/ITO/glass, (**b**) PANI/TiO_2_/ITO/glass, and (**c**) TiO_2_-PANI/ITO/glass.

An indium tin oxide (ITO) substrate (1 × 2 cm^2^) was cleaned in methanol using an ultrasonic bath for 10 min. The same process was repeated using deionized (DI) water. Then, the substrates were dried using nitrogen gas. Before the deposition process, the substrates were covered with Kapton tape leaving a sensing area of 1 × 1 cm^2^. The role of Kapton tape is to create a mask for the area dedicated for further use as a connection area between SE and copper wire. Figure 10 shows the sample structure of the TiO_2_, TiO_2_–PANI bilayer composite, and TiO_2_–PANI composite thin films.

#### 4.2.1. TiO_2_ Thin Film

The TiO_2_ solution was prepared using titanium isopropoxide, Ti[OCH(CH_3_)_2_]_4_, as a precursor, glacial acetic acid (GAA) as the stabilizer, Triton X-100 as the surfactant, and ethanol absolute as the solvent. The solution was stirred on a digital hotplate stirrer for 1 h at room temperature to allow all the reactions to complete. The thin film was spin-coated on an ITO substrate with a spin speed of 3000 rpm for 60 s. Ten drops of the TiO_2_ solution were utilized during the deposition process. The samples were then dried in ambient air for 100 °C for 10 min before being annealed at 400 °C for 15 min.

#### 4.2.2. TiO_2_–PANI Bilayer Composite Thin Film

The TiO_2_–PANI bilayer composite thin film consists of a TiO_2_ thin film as the first layer (bottom layer) and a PANI thin film as the second layer (upper layer). The TiO_2_ thin film was prepared using the same process as above. Then, the PANI solution was prepared by dissolving the emeraldine base PANI (0.1 wt%) in methanol and stirred for 1 h. The PANI solution was dropped ten times on a TiO_2_ thin film during the deposition process. The spin speed was set to 3000 rpm in 60 s. The samples were dried at 150 °C for 10 min.

#### 4.2.3. TiO_2_–PANI Composite Thin Film

The composite solution of TiO_2_–PANI was prepared by mixing both solutions of TiO_2_ and PANI. The emeraldine base PANI (0.1 wt%) was dissolved in methanol and stirred for 1 h. The TiO_2_ solution was prepared using the same process as above. TiO_2_ and PANI solutions were mixed with a ratio of 2:1 and stirred for another 15 min. Then, the samples were deposited on an ITO substrate with a spin speed of 3000 rpm for 60 s. The samples were dried at 150 °C for 10 min.

### 4.3. EGFET Measurement Setup

**Figure 11:**
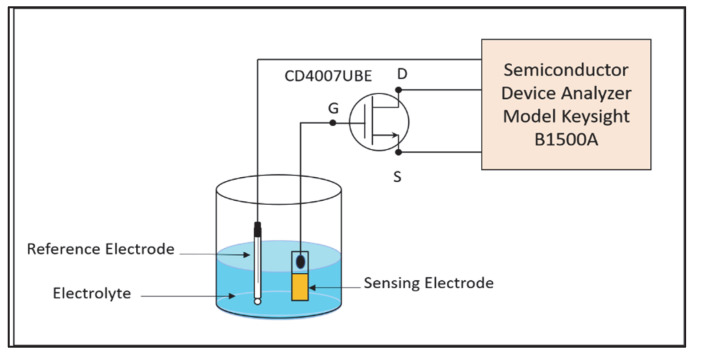
EGFET Measurement Setup.

The pH and nitrate detection was measured using the EGFET measurement as shown in Figure 11. Before the measurement was carried out, the connector area that connects the SE with the Cu wire was isolated using epoxy resin in order to avoid the Cu wire from coming into contact with the measured solution. Then, the other end of the Cu wire was connected to the gate of a commercialized metal–oxide–semiconductor field-effect transistor (MOSFET) CD4007UBE using copper wire. A commercialized reference electrode (RE) (RE-1B Ag/AgCl, Metrohm) was used to keep a constant potential during measurement. Both the MOSFET and RE were connected to a Semiconductor Devices Analyzer Keysight B15000A.

#### 4.3.1. pH Detection

The pH buffer solutions (pH 2, 4, 7, 10, and 12) were purchased from Sigma Aldrich. The experiments were carried out from neutral pH values and then propagated to basic and acidic directions to avoid destruction of the electrode in high basic or acid solutions.

#### 4.3.2. Nitrate Detection

Nitrate standard solutions (Thermo Fisher Scientific) were used for testing these sensing electrodes. Serial dilution of the ${\mathrm{NO}}_{3}^{-}$-N standard of concentration 100 ppm was carried out to obtain 10, 15, 20, 25, and 30 ppm. A nitrate ionic strength adjuster (Nitrate ISA, Orion ionplus Application solution) was added to the analyte with a certain ratio specified by the supplier manual.

## Figures and Tables

**Table 1:** EDX results of three thin films.

Element	TiO_2_ Thin Film (at%)	TiO_2_–PANI Bilayer Composite (at%)	TiO_2_–PANI Composite (at%)
C	1.8	0.1	0.7
O	37.4	39.6	39.0
In	18.5	17.9	18.9
Sn	2.2	1.5	1.6
Si	30.5	30.4	29.2
Ti	0.4	0.4	0.3
Na	3.6	3.4	3.4
Ca	4.1	5.1	4.9
Mg	0.8	1.0	1.2
Al	0.7	0.7	0.9

## Data Availability

Data will be available upon reasonable request.
